# The Effect of Hip Bracing on Gait in Patients with Medial Knee Osteoarthritis

**DOI:** 10.1155/2012/240376

**Published:** 2012-07-25

**Authors:** David Wallace, Christa Barr

**Affiliations:** Department of Physical Therapy, Quinnipiac University, Hamden, CT 06518, USA

## Abstract

*Objective.* Impaired hip motion has been associated with heightened medial knee joint loading in patients with knee osteoarthritis (OA). A hip external rotation strap designed to pull the femur into external rotation and abduction may serve as one protective mechanism. The primary aim of our study is to determine if the strap decreases medial knee joint loading during level walking in people with knee OA. *Design.* This study is a single-day repeated measures design. *Methods.* 15 volunteers with medial knee OA underwent motion analysis data collection during two randomly assigned walking conditions: (1) wearing the strap and (2) control (no strap). Primary outcome measures were peak pelvis, hip and knee joint motions, and torques. These outcomes were averaged across five trials for each condition. *Results.* Hip abduction (*P* < 0.01), trunk lean towards the stance limb (*P* = 0.04) and pelvic tilt (*P* = 0.02) significantly increased with the strap versus control trials. Knee adduction loading did not significantly change with the strap (*P* = 0.33). *Conclusion.* The use of the hip external rotation strap resulted in angular changes at the hip and pelvis which may be beneficial for patients with medial knee osteoarthritis.

## 1. Introduction

Knee osteoarthritis (OA), the most common form of arthritis, is projected to affect 67 million Americans by 2030 [1, 2]. This disease is associated with decreased function and impaired mobility [2]. Most often, patients choose to pursue physical therapy intervention prior to undergoing surgery. Currently-accepted physical therapy intervention for knee OA typically emphasizes quadriceps strengthening [3, 4]. However, recent research has called into question the effectiveness of quadriceps strengthening in slowing disease progression [4–6].

Since in vivo measurements of medial knee load are an unreasonable outcome measure in arthritis research with a volunteer subject population, the external knee adduction moment is used as a reliable estimate for medial knee compartment load [7, 8]. Many studies have established that an increased peak knee adduction moment during stance, especially an increased first peak, is characteristic of people with knee OA [9–11]. It is assumed that decreasing the knee adduction moment represents a reduction in medial knee joint loading and therefore, may have a beneficial effect on knee OA disease progression [8, 12]. 

Recent research has highlighted the importance of the kinematic chain when considering physical therapy treatment interventions for people with knee OA. Because the knee does not function in isolation from the rest of the lower extremity, the hip, pelvis, and trunk may play a role in influencing medial knee load during ambulation [7, 8]. Increased mediolateral trunk sway has been shown to decrease the knee adduction moment in healthy individuals during level gait [12]. Müundermann et al. [11] found that many people with knee OA had increased lateral sway of the trunk. The study reported that patients with severe knee OA demonstrated lower hip adduction moments in stance and proposed that this was due to limited hip abductor strength necessary to maintain the altered trunk position [11]. Hip abductor muscle weakness would cause the contralateral pelvis to drop in a trendelenburg fashion. The pelvic drop would result in the group reaction force being positioned more medially, further away from the knee joint center, thus increasing the knee adduction moment [10]. Multiple studies have reported that people with knee OA demonstrate significant strength deficits in the hip musculature, particularly of the hip abductors compared to age matched controls [13–15]. Furthermore, impaired hip mechanics have been associated with increased medial compartment knee loads [11, 16].

Recently, hip muscle strengthening has been proposed as a mechanism of stabilizing the pelvis in the frontal plane resulting in reduced medial knee loads [8, 17]. However, research has failed to demonstrate that hip muscle strengthening will significantly reduce medial knee joint load in people with knee OA [14, 15, 18]. Bennell et al. [18] reported that hip muscle strengthening improved function and reduced pain without any significant change in joint loading. A possible explanation for these findings is that people with knee OA may have developed compensations during walking where they utilize altered lower extremity mechanics to avoid pain. Muscle strengthening alone, in this population, may not be enough to alter these compensatory mechanics during ambulation. Despite the ability to improve hip abductor strength in people with knee OA, because of painful symptoms, improved strength alone may not eliminate the compensatory gait pattern in this cohort resulting in unaltered knee joint loading [14, 15, 18].

Valgus bracing is also a popular treatment for people with knee OA even though studies have demonstrated mixed results in regards to compliance and joint loading [19–22]. These braces tend to be large and bulky resulting in patient noncompliance. One study noted a limitation that most patients did not wear the brace consistently enough to adhere to the treatment protocol [23]. Valgus bracing does seem to reduce pain and improve function in some studies, but it does not appear to decrease medial compartment loading or slow disease progression on a consistent basis [20, 21, 23]. 

Bracing of the femur and pelvis may provide the pelvic stability needed to alter the compensatory mechanisms during ambulation. A strap designed to pull the femur into external rotation and abduction mimicking the natural action of the gluteus medius and gluteus maximus may serve as one mechanism to help decrease medial knee joint loads in people with knee OA. We propose that this strap could be used as an adjunct to therapy including hip and lower extremity muscle strengthening for knee OA. The hip strap may be a superior option compared to valgus bracing because it works with the natural pull of the hip musculature to alter lower extremity mechanics rather than forcing the knee into a valgus position. This type of brace is not bulky and can be worn under clothes, which may improve patient compliance with the treatment protocol.

The primary aim of our study is to determine if a hip external rotation strap will improve tibiofemoral joint mechanics and significantly reduce medial knee joint loading during level walking in people with knee OA.

## 2. Methods

### 2.1. Subjects

Recruitment of participants consisted of advertisements placed in local newspapers. Prior to inclusion, all persons were screened over the phone by a trained research assistant. Inclusion criteria consisted of the following: (1) greater than 18 years of age, (2) diagnosis of medial knee OA from a licensed physician, (3) Kellgren-Lawrence scale grade of 2 or greater, (4) complaints of pain for more than half of the days in a month, and (5) ability to ambulate 150 feet without an assistive device. Subjects were excluded from the study if they had a history of lower extremity injury or disease other than OA within the past two years or if they had a history of neurologic event or injury to the spine. Intra-articular injections to minimize the symptoms of knee OA within the past 6 months were also grounds for exclusion. If the subject had medial knee OA bilaterally, the most painful side was included in the study. All persons who met the inclusion criteria and signed an informed consent form approved by the University's Institutional Review Board were included in the study.

### 2.2. Numeric Pain Rating

Volunteers filled out a pain questionnaire based on the magnitude of their pain in their involved knee suffered over the previous 24 hours. The questionnaire, filled out on the day of data collection, included 6 items: (a) pain at rest, (b) pain walking on a level surface, (c) pain walking up and down stairs, (d) pain at worst, and (e) pain at best. A visual analog scale (VAS) was provided for each questionnaire item where zero indicated “no pain at all” and ten indicated “the worst pain possible”. Subjects were asked to mark on the line what level their knee pain was during each activity. Pain during ambulation was later used to group volunteers for further data analysis.

### 2.3. The Hip External Rotation Strap

**Figure 1:**
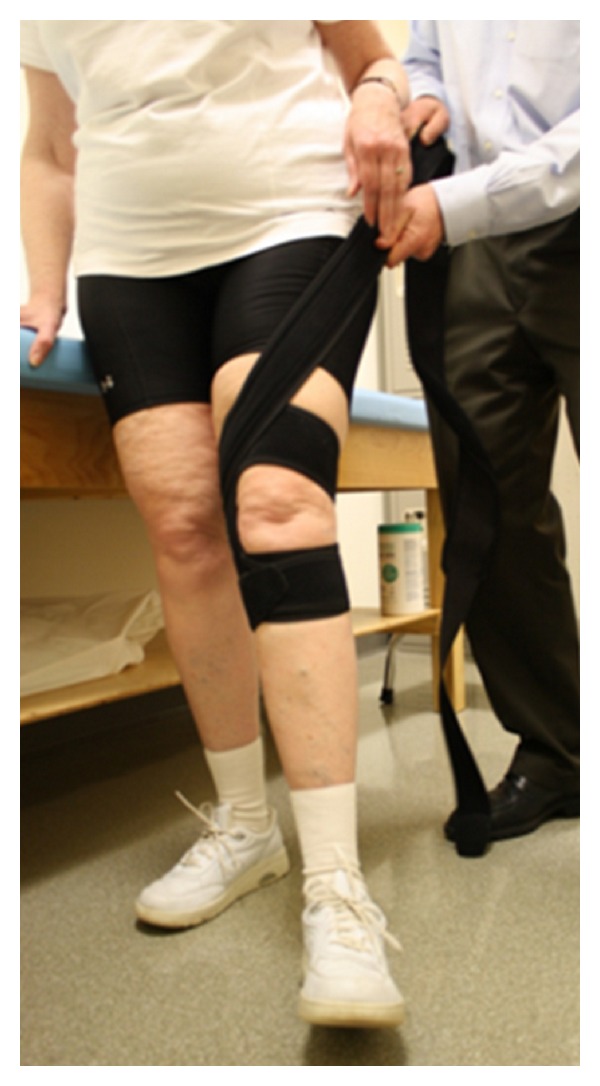
Hip external rotation strap.

The brace (S.E.R.F. Strap, Don Joy, Vista, CA) used in this study consisted of a thin neoprene strap that slides onto the lower leg just distal to the knee wraps around the femur in a medial-to-lateral and distal-to-proximal direction and is anchored by wrapping around the pelvis (Figure 1). The strap is designed to pull the femur into external rotation and abduction and stabilize the pelvis in the frontal plane. 

### 2.4. Equipment

Gait analysis was conducted in a Motion Analysis Lab. Reflective markers were applied bilaterally to various boney prominences in the upper and lower extremities and thorax for the gait trials including: acromion, lateral epicondyle of the humerus, capitate, anterior superior iliac spine, sacrum, calcaneus, and head of the third metatarsal as well as an offset marker on the right scapula. Four clusters of three markers each were placed bilaterally on the midshaft of the femur and tibia. Each of these clusters was secured with Coflex (Andover Healthcare, Inc., Salisbury, MA). Additionally, markers at the medial and lateral femoral epicondyles and medial and lateral malleoli were included during a static standing trial. A static trial, in the T-position with arms abducted to 90 degrees, was conducted prior to ambulation to establish location of reflective markers with respect to the lab coordinate system. After the static trial was collected, makers on the femoral epicondyles and malleoli were removed. Three-dimensional kinematic data was collected using 10-camera infrared motion capture system (Motion Analysis Corp, Santa Rosa, CA, USA) recorded at 120 Hz. Additionally, three-floor mounted force plates collected ground reaction force data (AMTI, Boston, MA, USA) at a sampling rate of 2400 Hz. Walking velocity was measured and recorded using an infrared timing light system (Equine Electronics, Peculiar, MO, USA) for each trial. This system consisted of a photogate system with two infrared beams from an IR emitter to an IR receiver providing output to a RF receiver and a stopwatch. This system recorded gait speed as subjects ambulated across the walkway by starting when the subject passed through the first beam and stopping when they passed through the second beam.

### 2.5. Walking Trials Data Collection

Prior to data collection volunteers donned the hip external rotation strap and were allowed to walk for 5 to 10 minutes to acclimate to its line of pull. The strap was removed and a 6-second static trial was conducted in which the volunteers were asked to stand in the T-position as still as possible. Next, subjects ambulated over a 10 meter walkway during two randomly assigned conditions: (1) wearing the hip external rotation strap and (2) without the strap (control). A total of five successful trials were collected in which the test limb landed on the force platform for the stance phase at each subject's self selected and habitual pace (±5%) for each condition. Rests were provided to the subjects as needed throughout the data collection process. Volunteers were required to wear tight fitting shorts, a t-shirt and were provided sneakers during gait testing for both conditions.

### 2.6. Data Analysis

Walking trials were processed by a trained research assistant for further analysis. Cortex software (Motion Analysis Corporation, Santa Rosa, CA, USA) was used to capture, digitize, and calculate the three-dimensional trajectories of the reflective markers during ambulation for each trial. The 22 anatomical markers were used to create coordinate systems and calculate three-dimensional motion for the trunk, pelvis, hip, knee, ankle, and foot. This data then underwent further processing through OrthoTrak software (Motion Analysis Corporation, Santa Rosa, CA) to quantify kinematic and kinetic data and identify events such as heel strike and toe off within each gait cycle. Three-dimensional joint moments were calculated using inverse dynamics and a lower extremity model as linkage of three rigid structures representing the thigh, shank, and foot. All joint moments were normalized to the subject's height and weight and were expressed as internal moments. Knee, hip and pelvic moments, and angles were normalized to 100% of stance and averaged across five trials for the braced and unbraced conditions. Paired *t*-tests were utilized for comparisons between the braced and unbraced conditions. Using a bonferroni correction, alpha was maintained at 0.05.

## 3. Results

Fifteen subjects (10 female, 5 male, mean age 60.5 ± 7.8 years, and mean body mass index 29.8 ± 3.9) participated in this study.

### 3.1. Kinematics

**Figure 2:**
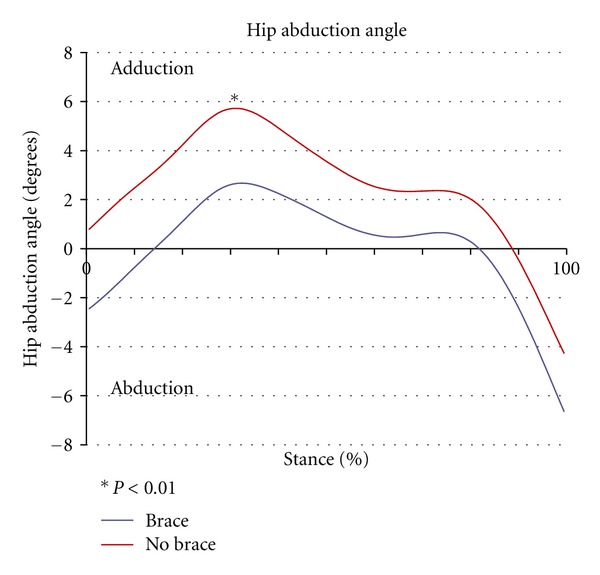
Hip abduction angle (degrees) in the braced versus control (no braced) conditions versus 100% of stance.

**Figure 3:**
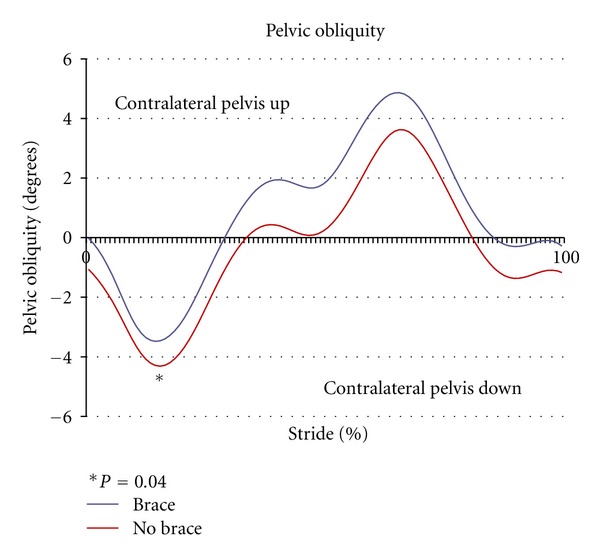
Pelvic obliquity (degrees) in the braced versus control (no brace) conditions versus 100% of stride. Positive pelvic obliquity represents contralateral ASIS superior and negative pelvic obliquity represents contralateral ASIS inferior.

**Figure 4:**
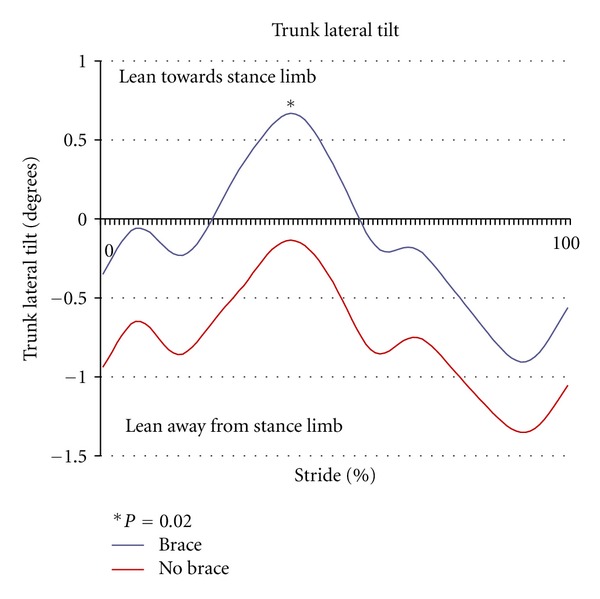
Trunk lateral tilt (degrees) in the braced versus control (no brace) conditions versus 100% of stride. Positive trunk lateral tilt represents lean towards the stance limb and negative trunk lateral lean represents lean away from the stance limb towards the swing limb.

Walking with the strap significantly increased the peak hip abduction angle during stance compared to the control condition (*P* < 0.01) (Figure 2). By limiting contralateral pelvic drop on the swing side in the frontal plane, wearing the strap significantly increased peak pelvic tilt in stance compared to control trials (*P* = 0.02) (Figure 3). Subjects also had significantly greater trunk lean angles when walking with the strap (*P* = 0.04) (Figure 4). There were no significant differences in sagittal plane angles at the hip or knee between the brace and unbraced condition (*P* > 0.05) as seen in Table 1. In addition, toe out angles were not significantly different between groups (*P* = 0.22) (Table 1). 

### 3.2. Kinetics

The use of the strap did not significantly decrease peak hip or knee moments versus the control trials (*P* = 0.33) as seen in Table 2. There was no significant change in the hip adduction moment with the strap versus control trials (*P* = 0.41). In addition, there were no statistically significant differences in hip or knee moments in the sagittal or transverse planes (*P* > 0.05) (Table 2).

Pain appeared to be an important factor to consider when evaluating the strap effectiveness. For further analysis, subjects were grouped according VAS pain severity reported during ambulation: (1) <1/10 or no pain, (2) 1–4/10 or minimal pain, (3) 4–7/10 or moderate pain, and (4) >7/10 or maximal pain. 

**Figure 5:**
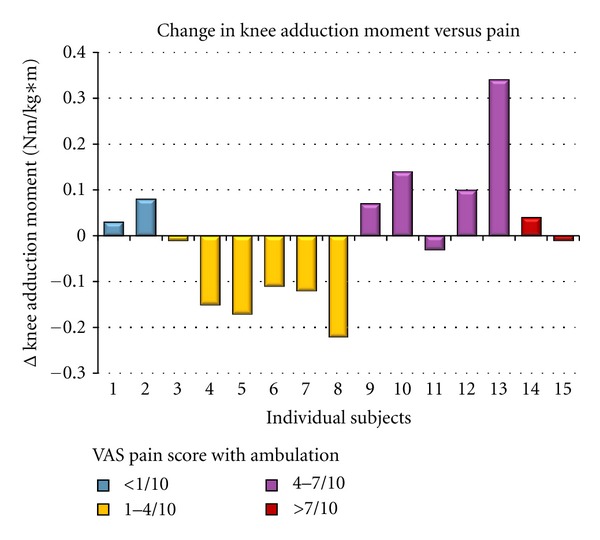
Change (from control to braced condition) in individual external knee adduction moment versus pain with ambulation for each individual subject. The subjects are grouped according to pain severity. Orange represents <1/10 pain or no pain, yellow represents 1–4/10 pain or minimal pain, green represents 4–7/10 pain or moderate pain and red represents >7/10 pain or maximal pain. *x*-axis represents individual subject pain reports.

 Upon visual comparison, it appears that 6 volunteers with minimal pain, or between grades 1–4 out of 10 during ambulation as reported on the VAS, experienced decreases in peak knee adduction moment during the braced condition by an average of 13%. In the patient subgroup with moderate to maximal pain (*n* = 7), the knee adduction moment actually increased in 5 of these 7 volunteers. The peak knee adduction moment for the subgroup with moderate to maximal pain increased an average of 6%. Individual results for changes in knee adduction moment are reported in Figure 5 grouped by pain with ambulation.

## 4. Discussion

These findings support our first hypothesis; using the strap resulted in changes in angles at the hip, pelvis, and trunk that may be beneficial for individuals with knee OA. Previous studies have examined the role of proximal mechanics during ambulation in this population and the effect of frontal plane trunk motion on knee joint loading [16, 24, 25]. Frontal plane trunk lean towards the stance limb has been suggested as a compensatory mechanism in people with knee OA that may play a role in decreasing knee adduction moment, thereby alleviating compressive loads across the medial compartment in stance [12, 16]. Mündermann et al. [12] reported that peak knee adduction moment could be reduced by up to 65% in normal subjects by increasing trunk lean towards the stance limb by 10 ± 5°. 

The use of the strap also increased peak pelvic tilt in the frontal plane suggesting that the strap might reduce unwanted pelvic as well as trunk motion. Linley et al. [10] reported that the pelvis and trunk both tilt toward the stance limb in people with knee OA who employed the compensation of increased trunk lean. In our study, when wearing the brace, there was an increased trunk lean towards the stance limb as well as increase in hip abduction angles and increased lateral pelvic tilt. These data suggest that the hip strap should enhance proximal compensatory mechanisms that have been proposed as a means to decrease knee adduction moments [12, 16, 24, 25]. 

Despite the significant changes in trunk and hip frontal plane kinematics, there was no translation to significant improvement in peak knee adduction moments in our study. Therefore, we were not able to support our second hypothesis that the strap would decrease knee adduction loading. Lack of change in the external knee adduction moment in the braced versus control condition found in this study is similar to findings reported in other studies [20, 26, 27]. These studies specifically evaluated valgus bracing and found that it had no effect on peak knee adduction moment during stance. Specifically, Gaasbeek et al. [26] reported that knee valgus bracing did not significantly reduce knee adduction moment versus same day control trials after wearing the brace for six weeks. Interestingly, the knee adduction moment was significantly decreased in the braced condition at six weeks versus the unbraced condition measured at zero weeks. These findings suggest that the valgus brace may have changed gait mechanics over the six week period but this did not result in significant decrease in knee joint loading at the six week mark. On the contrary, there are several other studies that did report differences in knee adduction moment during knee bracing in this population and concluded that bracing may be an effective method to decrease medial knee joint load [21, 22, 28]. Pollo et al. [21] utilized an adjustable valgus brace with a strain gauge and found that the brace significantly decreased average medial knee joint load, calculated using an analytical model, but did not influence net peak knee adduction moment. Clearly, there is some ambiguity in the literature concerning the effectiveness of knee bracing for osteoarthritis in decreasing knee joint load. Although this study utilized a hip and pelvic strap in the effort to influence knee joint mechanics, to our knowledge no other studies have been conducted with this specific type of brace in a population with knee OA. 

The hip abductor muscles function to maintain pelvis frontal plane alignment as well as provide stability at the hip during single leg stance [13]. Weak hip abductors often cause the contralateral pelvis to drop in single limb stance resulting in hip adduction and internal rotation [8, 17]. Hip adduction causes the center of mass to shift away from the joint center increasing the distance between the ground reaction force and the knee joint axis resulting in increased knee adduction joint moments [7]. Subsequently, the hip abductors may have an important role in the treatment of people with medial knee OA. Only a few studies have examined the effect of hip and pelvic motions on knee joint loads [8, 10, 12, 16]. Hip abductor muscle contraction is the largest contributor to the internal hip abduction moment [13]. Chang et al. [8] suggested that peak internal hip abduction moment was greater in knees with OA that did not progress compared to knees with progressing OA over a period of 18 months. These results suggest that increased internal hip abduction moment may serve as a protective mechanism for knee OA progression.

Although no significant differences in external knee adduction moment were found, there did appear to be a subgroup within our study for which the strap may have been more effective. It is our hypothesis that these unexpected outcomes are a result of the hip strap altering proximal hip, trunk and pelvic mechanics during ambulation. Our results of lack of significant change in knee adduction moment in braced versus control trials might be explained in that patients in the moderate-to-maximal pain subgroup may have already developed proximal compensatory mechanisms designed to reduce joint loading. Therefore, bracing did not provide an additive effect to further decrease the knee adduction moment. This is supported by Hunt et al. [25] confirming that lateral trunk lean in patients with knee OA was significantly positively correlated with WOMAC pain scores and OA disease severity. In addition, we suspect that the patients with pain greater than 7/10 during level walking may have also employed additional compensations during gait prior to bracing such as increased toe out angle and limiting knee flexion during stance [29, 30]. 

Interestingly, patients with minimal pain during level walking may have not yet developed compensatory strategies and therefore might have benefited from the altered proximal mechanics afforded by the hip brace. Although pain subgroups contained only a small number of subjects in each group, it appears as though more research needs to be done considering pain in relation to knee OA intervention and physical therapy treatment strategy. In addition, our study did not allow patients to wear the brace for a period of days or weeks before data collection and this could have had an impact on gait mechanics, neuromuscular activation, and knee moments. It may have been beneficial to allow patients to wear the strap for at least two weeks to acclimate to the pull and help normalize the gait pattern prior to motion analysis as is consistent with other studies of this nature [28, 31]. 

Overall, our results suggest that femoral strapping can effect hip and knee kinematics and might possibly decrease knee joint loading in people with mild symptoms of pain. The strap may be a potentially beneficial adjunct to therapy and conservative treatment, especially in the population with mild to moderate pain during ambulation. However, further research should be conducted to establish a longitudinal relationship between pain, brace effectiveness, and proximal compensatory mechanics during ambulation of people with knee OA.

## Figures and Tables

**Table 1:** Peak kinematic data: brace versus no brace (mean ± standard deviation) (degrees).

Variable	No brace	Brace	*P* value
Peak hip flexion angle	35.7^°^ (9.8)	35.3^°^ (10.8)	0.43
Peak hip ER angle	8.1^°^ (11.6)	9.4^°^ (15.0)	0.56
Peak knee flexion angle	31.6^°^ (9.5)	30.2^°^ (12.0)	0.46
Peak knee abduction angle	3.8^°^ (3.4)	3.6^°^ (3.4)	0.44
Peak toe out angle	−0.9^°^ (10.8)	−1.7^°^ (12.2)	0.22

**Table 2:** Peak kinetic data: brace versus no brace (mean ± standard deviation) (Nm/kg∗m).

Variable	No brace	Brace	*P* value
Peak hip flexion moment (Nm/kg∗m)	5.1 (10.6)	5.3 (11.3)	0.39
Peak hip adduction moment (Nm/kg∗m)	2.7 (1.1)	2.8 (1.2)	0.41
Knee flexion moment (Nm/kg∗m)	4.2 (10.7)	4.4 (11.3)	0.62
Knee adduction moment (Nm/kg∗m)	1.5 (0.8)	1.5 (0.9)	0.33
